# Correlation of NQO1 and Nrf2 in Female Genital Tract Cancer and Their Precancerous Lesions (Cervix, Endometrium and Ovary)

**DOI:** 10.14740/wjon931w

**Published:** 2015-06-12

**Authors:** Nisreen Abdel Tawab Abdel Gaber Osman, Nehad M. R. Abd El-Maqsoud, Saad Abdelnaby A. El Gelany

**Affiliations:** a Department of Pathology, Faculty of Medicine, Minia University, Egypt; b Department of Obstetrics and Gynecology, Faculty of Medicine, Minia University, Maternity Hospital, Egypt

**Keywords:** Cervical carcinoma, Endometrial carcinoma, Ovarian carcinoma, NQO1, Nrf2

## Abstract

**Background:**

NAD (P) H/quinone oxidoreductase 1 (NQO1) is a metabolizing enzyme that detoxifies chemical stressors and antioxidants. Nuclear factor erythroid 2-related factor 2 (NrF2) is an important transcriptional activator involved in the cellular defense mechanisms against oxidative stress.

**Methods:**

The immunohistochemical expression of NQO1 and Nrf2 in 80 cervical, 80 endometrial and 100 ovarian specimens with different lesions was studied. Then we study the relation of both NQO1 and Nrf2 expression and clinicopathological features of carcinoma cases.

**Results:**

Immunohistochemical stain showed that NQO1 and Nrf2 were highly expressed in carcinoma compared with normal and precancerous lesions. Significant positive correlations were found between the mean expression of NQO1 and Nrf2 in different lesions. Moreover, there was significant correlation between the high level of NQO1 and Nrf2 expression and high tumor grade in cervical and endometrial carcinoma cases. Nrf2 expression was significant with advanced stage in endometrial and ovarian carcinomas.

**Conclusions:**

NQO1 and Nrf2 might be new biomarkers for early diagnosis and prognostic evaluation as well as being targets for therapy in patients with tumors in female genital tract.

## Introduction

Cancers of the female reproductive system include cervical, endometrial and ovarian cancers, which are relatively common and cause significant morbidity and mortality worldwide, whereas vulvar, vaginal and fallopian tube cancers are very rare [1]. Cervical cancer is the third most common cancer in women worldwide and the seventh most common cancer overall. Its overall mortality incidence ratio is 52% [2]. Endometrial cancer is the sixth most common cancer in women worldwide. Its incidence and mortality rates are higher in more developed regions and lowest rates occurring in Asia and Africa. Overall, the mortality incidence ratio of endometrial cancer is 26% [3]. Cancers of the ovary constitutes the eighth most common cancers among women worldwide with mortality incidence ratio of 62% [2]. In Middle Egypt with regional registry in Minia, the incidence of cervical, endometrial and ovarian cancer is 1.06%, 0.67% and 3.75% respectively of cancer sites in females [4]. Understanding the mechanisms of carcinogenesis in female reproductive organs could contribute to early detection, and will be helpful in the prevention and treatment of these cancers.

NAD (P) H/quinone oxidoreductase-1 (NQO1), also known as DT-diaphorase, is a cytosolic enzyme that uses NADH or NADPH as substrates to catalyze the two-electron reduction of quinones and related compounds, and it is encoded by a gene located on chromosome 16q22 [5]. In normal cells, NQO1 protects cells against oxidative stress, as well as against carcinogenesis by stabilization of the p53 tumor suppressor [6]. However, studies on NQO1 expression in cancer have been contradictory. On the one hand, NQO1 is induced along with a battery of defensive genes that provide protection against different stresses to prevent organs from undergoing carcinogen-induced tumorigenesis. On the other hand, reductive activation of environmental carcinogens including heterocyclic amines by NQO1 could contribute to carcinogenesis. Also, the disruption of the NQO1 gene or genetic polymorphism increased the risk of chemical-induced toxicity and cancers [7]. Comparing normal and malignant tissue, NQO1 was reported to be up-regulated in malignant tissue of the pancreas, interlobular biliary epithelial cells, breast and lung [8-11] and down-regulated in tumors of the kidney and esophagus [12, 13]. In addition, the high level of NQO1 expression in various tumors in combination with its ability to reduce many quinine-containing antitumor drugs has drawn attention to NQO1 as a potential molecular target in cancer treatment [14]. The molecular mechanism of NQO1 responsible for tumors of the female reproductive system progression remains unclear, and additional studies are needed to understand its role in female tumorigenesis.

The transcription factor, nuclear factor erythroid 2-related factor 2 (Nrf2) is a nuclear transcription factor maintaining intracellular redox homeostasis that induces transcription of a variety of genes through binding to the antioxidant response element in target gene promoters [15, 16]. It is induced in response to various agents at the transcriptional level. In addition, more than 200 gene products including an antioxidative enzyme NQO1 are under the transcriptional control for Nrf2 [17]. Therefore, activation of Nrf2 confers protection against cancer [18]. Furthermore, the beneficial effects of many chemopreventive compounds rely on the activation of the Nrf2-mediated antioxidant response through inhibition of Nrf2 degradation [19]. Mutations and deregulation of Nrf2 expression levels have been identified in many cancers [18] and lead to chemoresistance to many chemotherapeutic drugs [20, 21].

To date, the association and correlation between NQO1 and Nrf2 expression in cancers of the female reproductive system have not been adequately studied. In this study, we aimed to analyze the expression and relationship between both markers in carcinomas of the cervix, endometrium, ovary and their precancerous lesions. Also, we studied their relation with clinicopathological parameters in carcinoma cases for better understanding their role in tumorigenesis.

## Materials and Methods

### Tissue specimens

Formalin-fixed and paraffin-embedded specimens were collected and prepared for this study from Minia University Hospital in collaboration with the cancer unit in the Obstetrics and Gynecology Department, Minia University between January 2008 and December 2014. The histology of all cases using hematoxylin-eosin (H&E) stained slides was reviewed. The histological grade was assessed according to the World Health Organization (WHO) classification standards [22]. Tumors were staged according to the pathologic tumor-node-metastasis (TNM) and FIGO classification according to the Union for International Cancer Control (UICC) criteria seventh Edition and WHO classification [23]. The clinicopathological data were obtained from the pathology reports of cases. The available data include patients’ age, tumor grade and stage.

Cervical specimens, include 10 non-neoplastic cervical tissues, 20 squamous intraepithelial lesion (SIL) (eight cases of low-grade squamous intraepithelial lesion (LSIL) and 12 cases of high-grade squamous intraepithelial lesion (HSIL)) and 50 squamous cell carcinomas (SCCs). All cervical tissue specimens were selected from punch biopsies, loop electrosurgical excisions, cone biopsies and hysterectomies.

Endometrial specimens included 10 cyclic endometrium (CE) (six cases were proliferative phase (PP) and four cases were secretory phase (SP)), 20 cases endometrial hyperplasia (EH) (eight cases without atypia, 12 cases atypical EH) and 50 were endometrial carcinoma (EC) specimens. CE and hyperplasia samples were obtained either by curettage or biopsy specimens. Hyperplasia specimens were evaluated according to WHO classification [22]. All EC patients had undergone total abdominal hysterectomy and bilateral salpingo-oophrectomy.

Ovarian specimens included 10 cases of normal ovarian tissues, 20 cases of benign ovarian tumors (12 cases serous and eight cases mucinous), 20 cases of borderline ovarian tumors (12 cases serous and eight cases mucinous) and 50 cases of ovarian carcinoma (35 cases serous and 15 cases mucinous carcinoma). Normal ovarian specimens from hysterectomy specimens resected for non-ovarian disease were used. The majority of patients with a diagnosis of primary ovarian cancer had undergone radical surgery (staging laparotomy) according to standard operating procedures with the primary objective of maximal tumor reduction.

### Immunohistochemistry

Paraffin-embedded sections on coated slides were used for staining. Sections were cut at 4 μm thick. Immunohistochemistry was performed using the DAKO LSAB kit (DAKO A/S, Glostrup, Denmark) as follows: slides were deparaffinized in xylene and rehydrated in a graded alcohol series. Antigen retrieval was achieved by microwaving in sodium citrate buffer at pH 6 for 10 min at 95 °C. Endogenous peroxidase was blocked with 0.3% hydrogen peroxide in methanol for 30 min. The slides were incubated with the mouse monoclonal primary antibody for NQO1 (A180, ab28947, Abcam, 1:200), mouse monoclonal primary antibody for human Nrf2 (IgG2a, ab89443, Abcam, 1:100) overnight in a humidity chamber at 4 °C overnight. Then samples were washed with rinse puffer (PBS) and biotinylated secondary antibody for 30 min at room temperature. Streptavidin was applied for 30 min at room temperature. Visualization was performed using 3,3’-diaminobenzidine (DAB) chromogen, and Mayer’s hematoxylin was used for counterstaining for 10 min. A section of breast carcinoma and lung carcinoma were used as a positive control for NQO1 and Nrf2 proteins respectively. A negative control was carried out by replacing primary antibodies with rise buffer on a section.

### Evaluation of immunohistochemistry staining

Five random fields of vision in each section were selected and analyzed. The positive area, which was shown in percentage (ratio of positive area to the whole visual field) was calculated.

### Statistical analysis

Data were analyzed using the statistical package for the Social Sciences version 17.0 (SPSS 17.0). Raw data were used to determine means, standard deviations (SDs) and ranges. Kruskal-Wallis test was used to compare markers expression in different groups followed by Mann-Whitney test which was used to compare expression between two groups. Pearson correlation was used to determine whether there was a positive or negative correlation between each examined marker and each histopathological entity. Kruskal-Wallis test was used to examine the correlation of NQO1, Nrf2 staining scores in relation to tumor grade and stage. ANOVA test was used to examine the correlation of NQO1, Nrf2 staining scores in relation to age. Statistical significance was set at P ≤ of 0.05.

## Results

**Figure 1:**
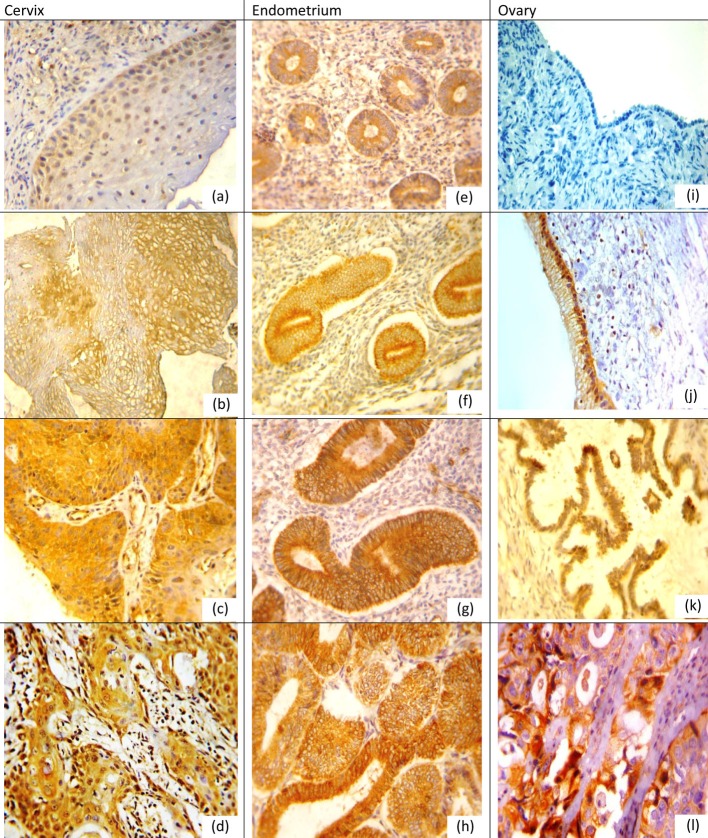
NQO1 protein expression in different lesions using IHC (× 400, DAB was used as the chromogen and hematoxylin as counterstain). (a) NQO1 protein was weak in normal cervical epithelium. (b) NQO1 protein staining was moderate positive in LSIL. (c) NQO1 protein showed diffuse and strong cytoplasmic-positive staining in HSIL. (d) NQO1 was strong positive in grade I cervical SCC. (e) NQO1 protein was weak in proliferative endometrium. (f) NQO1 was moderate positive in simple EH. (g) NQO1 was strong positive in atypical EH. (h) NQO1 was strong positive in EC. (i) NQO1 was negative in normal ovarian epithelium. (j) NQO1 was positive mucinous cystadenoma. (k) NQO1 was moderate positive in borderline serous tumor. (l) NQO1 was strong positive in ovarian carcinoma.

**Figure 2:**
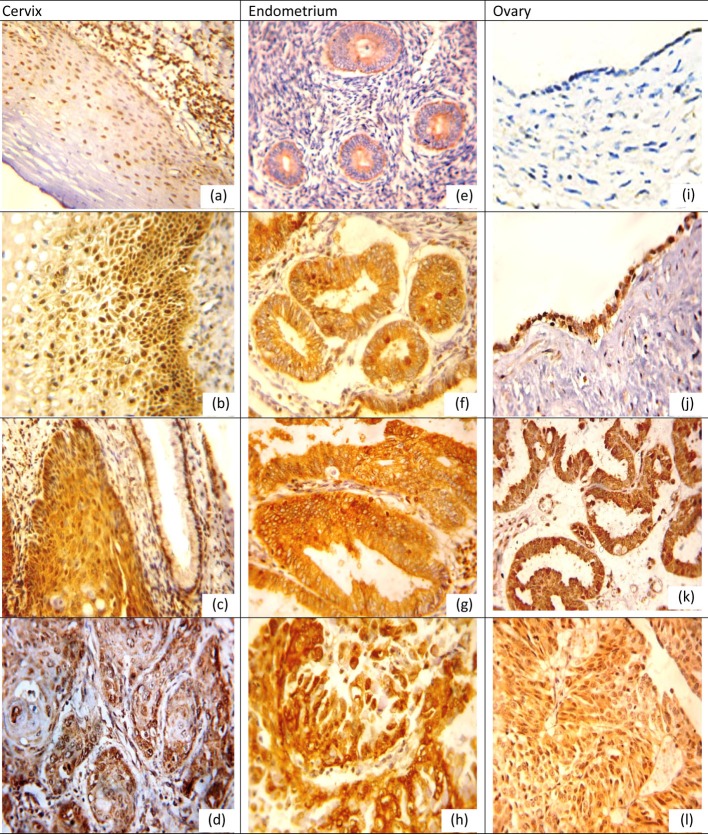
Nrf2 protein expression in different lesions using IHC (× 400, DAB was used as the chromogen and hematoxylin as counterstain). (a) Nrf2 protein was weak in normal cervical epithelium. (b) Nrf2 protein staining was moderate positive in LSIL. (c) Nrf2 protein showed moderate nuclear-positive staining in HSIL, but weak positive in adjacent normal cervical glands. (d) Nrf2 was strong positive in grade II cervical SCC. (e) Nrf2 protein was positive in proliferative endometrium. (f) Nrf2 was moderate positive in simple EH. (g) Nrf2 was moderate positive in atypical EH. (h) Nrf2 was strong positive in EC. (i) Nrf2 was negative in normal ovarian epithelium. (j) Nrf2 was positive serous cystadenoma. (k) Nrf2 was moderate positive in borderline serous tumor. (l) Nrf2 was strong positive in ovarian carcinoma.

Positive expression rates, mean values and SDs for NQO1 and Nrf2 in different lesions for organs examined are listed in Tables 1, 2. NQO1 expression was detected in the cytoplasm of examined tissue as shown in Figure 1 while Nrf2 was nuclear and cytoplasmic marker in cervical and ovarian tissue while in the endometrium its expression was mainly cytoplasmic with little nuclear expression in CE and EH, and nuclear expression was more pronounced in EC as shown in Figure 2.

**Table 1:** Positive Expression Rates, Mean Values and SDs for NQO1 in Different Lesions With Examined Sites

Site	Lesion	No.	% +ve	Mean ± SD	Min	Max	P-value among groups
Cervix*	Normal	10	20	3.70 ± 5.01	0	15	< 0.001
	SIL	20	45	18.35 ± 24.94	0	80	
	Carcinoma	50	76	44.34 ± 29.52	0	90	
Endometrium**	Cyclic endometrium	10	10	2.40 ± 3.30	0	10	0.001
	Hyperplasia	20	30	10.15 ± 15.61	0	50	
	Carcinoma	50	60	36.92 ± 31.94	0	85	
Ovary***	Normal	10	0	0	0	0	< 0.001
	Benign	20	20	6.30 ± 9.88	0	30	
	Borderline	20	25	12.40 ± 21.08	0	60	
	Carcinoma	50	56	28.30 ± 27.29	0	80	

Test of significance: Kruskal-Wallis, Mann-Whitney test. *Normal vs. SIL, P = 0.091; normal vs. carcinoma, P < 0.001; SIL vs. carcinoma, P = 0.004. **Cyclic endometrium vs. hyperplasia, P = 0.402; cyclic endometrium vs. carcinoma, P = 0.007; hyperplasia vs. carcinoma, P = 0.003. ***Normal vs. benign, P = 0.005; normal vs. borderline, P = 0.003; normal vs. carcinoma, P < 0.001; benign vs. borderline, P = 0.554; benign vs. carcinoma, P = 0.006; borderline vs. carcinoma, P = 0.076.

**Table 2:** Positive Expression Rates, Mean Values and SDs for Nrf2 in Different Lesions With Examined Organs

Organ	Lesion	No.	% +ve	Mean ± SD	Min	Max	P-value among groups
Cervix*	Normal	10	10	4.60 ± 5.42	0	15	0.001
	SIL	20	30	14.50 ± 18.20	0	60	
	Carcinoma	50	62	39.24 ± 29.25	0	85	
Endometrium**	Cyclic endometrium	10	10	4.30 ± 6.01	0	20	0.001
	Hyperplasia	20	35	12.20 ± 16.91	0	60	
	Carcinoma	50	64	39.82 ± 32.65	0	85	
Ovary***	Normal	10	0	0	0	0	< 0.001
	Benign	20	25	10.30 ± 14.50	0	50	
	Borderline	20	20	14.60 ± 19.33	0	60	
	Carcinoma	50	72	32.70 ± 29.44	0	85	

Test of significance: Kruskal-Wallis, Mann-Whitney test. *Normal vs. SIL, P = 0.155; normal vs. carcinoma, P = 0.002; SIL vs. carcinoma, P = 0.005. **Cyclic endometrium vs. hyperplasia, P = 0.350; cyclic endometrium vs. carcinoma, P = 0.009; hyperplasia vs. carcinoma, P < 0.005. ***Normal vs. benign, P = 0.015; normal vs. borderline, P = 0.003; normal vs. carcinoma, P < 0.001; benign vs. borderline, P = 0.529; benign vs. carcinoma, P = 0.005; borderline vs. carcinoma, P = 0.029.

### NQO1 expression in distinct tissue types

#### NQO1 expression in cervix

**Figure 3:**
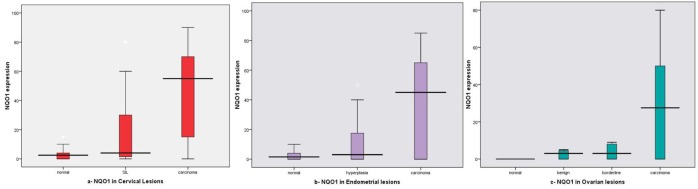
NQO1 expression box plots (a) in different cervical lesions; (b) in different endometrial lesions; (c) in different ovarian lesions. Horizontal lines in the boxes represent the median value of each group. The top and bottom edges of the boxes indicate the score values from 75th percentile and the 25th percentile respectively. Whiskers represent the highest and lowest values. The range is shown as a vertical line.

On studying the mean NQO1 expression in different lesions, we found increased expression in SIL (45%, mean ± SD: 18.35 ± 24.94) and cervical carcinoma (76%, mean ± SD: 44.34 ± 29.52) cases compared to normal tissue (20%, mean ± SD: 3.70 ± 5.01) and the difference between all examined groups was statistically significant (P < 0.001) as shown in Table 1, Figure 3a.

As regard SIL, we found in LSIL, NQO1, mean ± SD: 21.37 ± 3.11, while in HLSIL, NQO1: 16.33 ± 2.11, that was lower than in LSIL, and this difference did not reach a significant level (P = 0.885).

Regarding its mean expression in different lesions, statistically significant differences were seen between normal and carcinoma (P = 0.001) and between SIL and carcinoma (P = 0.004). In addition, no significant difference was noticed between NQO1 mean expression in normal and SIL (P = 0.091).

#### NQO1 expression in endometrium

We found increased mean expression rate in EH and EC was 30%, 60% with mean ± SD: 10.15 ± 15.61 and 36.92 ± 31.94 respectively, while decrease in CE (10%, mean ± SD: 2.40 ± 3.30), and the difference was statistically significant (P = 0.001) (Table 1, Fig.3b).

For EH, we found in typical EH and atypical EH the mean ± SD was 3.00 ± 3.33, 14.91 ± 18.75 respectively, and there were no statistically significant differences between both (P = 0.118).

Regarding its mean expression in different lesions, statistically significant differences between EC and EH, CE (P = 0.003, P = 0.007 respectively) were noted. There were no statistically significant differences between EH and CE (P = 0.402).

#### NQO1 expression in ovary

On studying the NQO1 mean expression in different lesions, we found increased expression in carcinoma cases (56%, mean ± SD: 28.30 ± 27.29) than other lesions (20%, mean ± SD: 6.30 ± 9.88 in benign; and 25%, mean ± SD: 12.40 ± 21.08 in borderline tumors), and the difference was statistically significant (P < 0.001) as shown in Table 1, Figure 3c.

Regarding NQO1 mean expression in different lesions, statistically significant differences were seen between normal and each benign, borderline and malignant tumors (P = 0.005, P = 0.003 and P < 0.001 respectively). Statistically significant difference was seen between benign and carcinoma (P = 0.006). No statistically significant difference was seen between benign and borderline (P = 0.554), between borderline and carcinoma (P = 0.076).

### Associations between NQO1 expression and clinicopathological data in carcinoma cases

Associations between clinicopathological data and NQO1 mean expression were summarized in Table 3.

**Table 3:** Associations Between NQO1 and Nrf2 Expression Scores and Clinicopathological Data in Carcinoma Cases

Organ	Clinicopathological parameter	No. of cases	NQO1		Nrf2	
			Mean ± SD	P-value	Mean ± SD	P-value
Cervix	Age					
	Mean	55.20 ± 6.54	44.34 ± 29.52	0.445	39.24 ± 29.25	0.526
	Grade					
	I	14	25.14 ± 33.26	0.045	25.14 ± 33.03	0.016
	II	20	26.80 ± 29.65		28.00 ± 29.11	
	III	16	33.50 ± 32.61		23.00 ± 23.59	
	Stage					
	I	8	40.62 ± 37.74	0.461	36.88 ± 32.28	0.594
	II	28	48.32 ± 29.80		35.11 ± 29.01	
	III	10	42.40 ± 25.57		48.50 ± 28.67	
	IV	4	28.75 ± 20.15		49.75 ± 29.89	
Endometrium	Age					
	Mean	51.90 ± 5.77	36.92 ± 31.94	0.802	39.82 ± 55.00	0.923
	Grade					
	I	16	17.25 ± 27.14	0.011	18.44 ± 28.61	0.002
	II	21	45.38 ± 31.39		48.48 ± 31.35	
	III	23	47.46 ± 28.99		52.15 ± 28.06	
	Stage					
	I	10	12.60 ± 23.98	0.082	13.40 ± 24.73	0.025
	II	13	40.69 ± 32.61		41.54 ± 31.31	
	III	19	42.00 ± 31.28		49.11 ± 33.18	
	IV	8	49.13 ± 30.82		48.00 ± 29.21	
Ovary	Age					
	Mean	55.74 ± 9.92	28.30 ± 27.29	0.734	32.70 ± 29.44	0.395
	Grade					
	I	10	22.70 ± 30.31	0.712	30.80 ± 37.00	0.576
	II	23	26.87 ± 26.72		29.26 ± 30.14	
	III	17	33.53 ± 27.02		18.47 ± 24.10	
	Stage					
	I	8	12.50 ± 21.66	0.167	16.88 ± 28.77	0.020
	II	15	22.53 ± 30.07		19.80 ± 31.06	
	III	15	33.47 ± 25.14		45.27 ± 25.06	
	IV	12	39.58 ± 25.71		43.67 ± 23.50	

Test of significance: Kruskal-Wallis, ANOVA tests. P-value < 0.05 is considered significant.

As regarding cervical carcinoma, a significant association between increased NQO1 mean expression and tumor grade (P = 0.045) was detected. No significant associations were noticed between its expression and either age or stage.

In EC, a significant association was noticed between increased NQO1 mean expression and tumor grade (P = 0.011). No significant associations were noticed between its expression and either age or stage.

Regards ovarian carcinoma, no significant associations were noticed between NQO1 mean expression and any clinicopathological data.

### Nrf2 expression in distinct tissue types

#### Nrf2 expression in cervix

**Figure 4:**
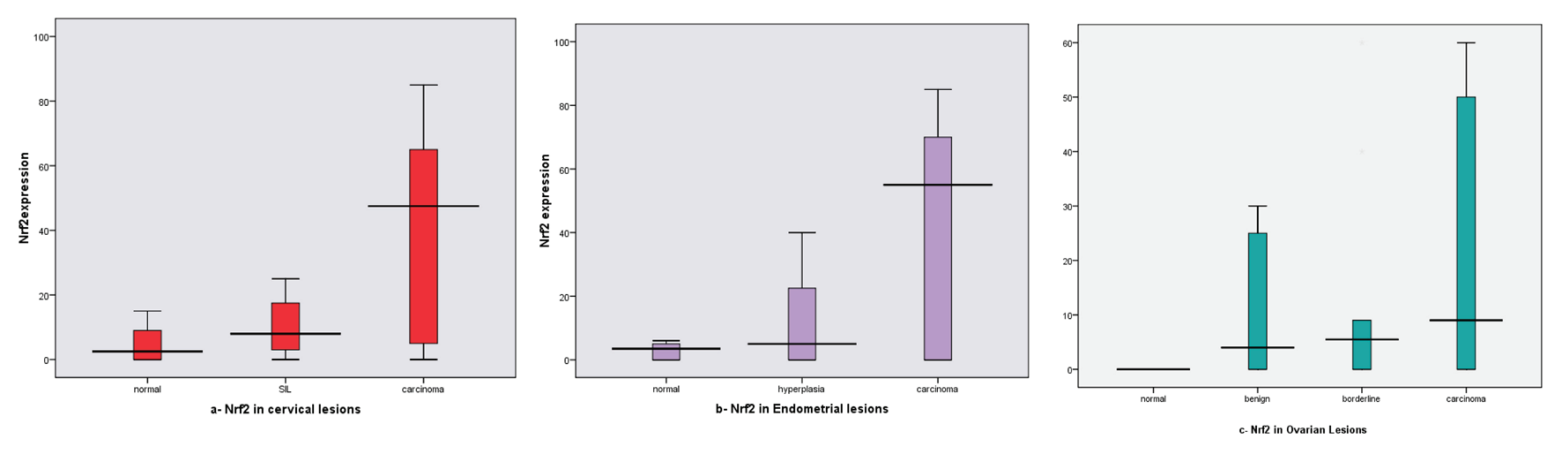
NrF2 expression box plots (a) in different cervical lesions; (b) in different endometrial lesions; (c) in different ovarian lesions. Horizontal lines in the boxes represent the median value of each group. The top and bottom edges of the boxes indicate the score values from 75th percentile and the 25th percentile respectively. Whiskers represent the highest and lowest values. The range is shown as a vertical line.

As regard Nrf2 expression in different lesions, we found increased mean expression from normal tissue (10%, mean ± SD: 4.60 ± 5.42) to SIL (30%, mean ± SD: 14.50 ± 18.20) and to cervical carcinoma (62%, mean ± SD: 39.24 ± 29.25). The difference between all examined groups was statistically significant (P = 0.001) as shown in Table 2, Figure 4a.

Regarding Nrf2 expression in SIL, we found that its expression in LSIL mean ± SD: 11.12 ± 14.21 and in HSIL 16.75 ± 20.37, and there was no statistically significant difference between both (P = 0.451).

Regarding its mean expression in different lesions, statistically significant differences between normal and carcinoma (P = 0.002) and between SIL and carcinoma (P = 0.005) were found. No significant difference was noticed between Nrf2 mean expression in normal and SIL (P = 0.155).

#### Nrf2 expression in endometrium

Nrf2 expression rate was increased in EH (35%, mean ± SD: 12.20 ± 16.91) and EC (64%, mean ± SD: 39.82 ± 32.65) than in CE (10%), and the difference was statistically significant (P = 0.001) as shown in Table 2, Figure 4b.

For EH, we found, in typical EH and atypical EH the mean ± SD was 10.25 ± 12.72, 13.50 ± 19.66 respectively, and there were no statistically significant differences between both (P = 0.603).

As regard Nrf2 mean expression in different lesions, statistically significant differences between CE and EH, EC (P = 0.009, P = 0.005 respectively). There were no statistically significant differences between CE and EH (P = 0.350).

#### Nrf2 expression in ovary

Nrf2 expression in different ovarian lesions, we noticed that increased mean expression in carcinoma (72%, mean ± SD: 32.70 ± 29.44) than other examined lesions (0% for normal; 25%, mean ± SD: 10.30 ± 14.50 for benign tumors; and 20%, mean ± SD: 14.60 ± 19.33 for borderline tumors), and the difference reached a significant statistical level (P = 0.004) (Table 2, Fig.4c).

Regarding its mean expression in different lesions, statistically significant differences were identified between normal and carcinoma (P < 0.001). Statistically significant differences were identified between benign and carcinoma (P = 0.005), and between borderline and carcinoma (P = 0.029). Similarly, statistically significant difference was seen between normal and benign and borderline (P = 0.015, P = 0.003 respectively). In addition, no statistically significant difference was seen between benign and borderline (P = 0.529).

### Associations between Nrf2 expression and clinicopathological data in carcinoma cases

Associations between clinicopathological data and Nrf2 mean expression were summarized in Table 3.

In cervical carcinoma, we found that a significant association between increased Nrf2 mean expression and high tumor grade (P = 0.016) was detected. No significant associations were noticed between its mean expression and age or stage.

For Nrf2 overexpression in EC, we found that a significant association between increased its mean expression and tumor grade and stage (P = 0.002, P = 0.025 respectively) was detected.

Regarding ovarian carcinoma, a significant association was noticed between increased Nrf2 mean expression and advanced tumor stage (P = 0.020). No significant associations were noticed between its expression and either age or grade.

### Correlations between immunohistochemical markers expression

Correlations between immunohistochemical markers expression in different cervical lesions were found. A significant positive correlation was noted between NQO1 and Nrf2 mean expression in all examined cases (r = 0.734, P < 0.001). A significant positive correlation was noted between NQO1/Nrf2 (r = 0.818, P < 0.001) in SIL. Similarly, a significant positive correlation was noted (r = 0.615, P < 0.001) in carcinoma. No significant correlations were noted between them in normal cervical tissue (r = 0.615, P = 0.051).

Correlations between immunohistochemical markers expression in different endometrial lesions were found. A significant positive correlation was noted between NQO1 and Nrf2 mean expression in all examined cases (r = 0.922, P < 0.001). A significant positive correlation was noted (r = 0.909, P < 0.001) in CE. Similarly, a significant positive correlation was noted (r = 0.637, P = 0.003) in EH. Also a significant correlation was in EC (r = 0. 925, P < 0.001).

Correlations between immunohistochemical markers expression in different ovarian lesions were found. A significant negative correlation was noted between NQO1 and Nrf2 (r = 0.741, P < 0.001) in all cases. Similarly, a significant positive correlation was noted in benign tumors (r = 0.623, P = 0.003) and in carcinoma (r = 0.740, P < 0.001). No significant correlations were noted in borderline tumors (r = 0.6432, P = 0.057).

## Discussion

NQO1 flavoprotein has been found to be expressed in many body tissues [9-11]. It is conceivable that NQO1 is primarily involved in protecting normal cells from oxidant stress. Such finding has led to the suggestion that NQO1 can be important in cancer chemoprevention. However, polymorphism in the NQO1 gene has been reported to be associated with an increased risk of various cancers such as breast [10], lung [11], gastric [24] and head and neck cancer [25].

In the present study, we found that staining of NQO1 is mainly localized in the cytoplasm and these observations were in agreement with previous studies [8-10, 26, 27]. NQO1 has positive cytoplasmic expression in 76%, 60% and 65% in cervical, endometrial and ovarian carcinoma respectively. Previous studies demonstrated that NQO1 immunopositivity rate ranged between 21% and 80% [10, 11, 28] in various tumors. We found increased NQO1 expression from normal tissue to SIL and cervical carcinoma and then from SIL to carcinoma. The difference between all examined groups was statistically significant. This finding was also observed in the endometrial and ovarian lesions. In a previous study, a strong positive rate of NQO1 protein expression in both SCCs (54.80%) and cervical intraepithelial neoplasia (CINs) (27.59% in CIN-1, 34.21% in CIN-2 and 40.74% in CIN-3) was significantly higher than in the normal cervix (4%). Interestingly, the strongly positive rate of NQO1 protein was slightly higher in well-differentiated SCC (43.75%) than in CIN3 (40.74%) [26]. In addition, previous results noted more NQO1 overexpression in carcinoma than in normal or precancerous lesions in breast [10], colon [27] and liver [28], and the difference reached a significant level. Our results with previous results indicate that NQO1 up-regulation may be an early event in cancer progression. These findings suggest that NQO1 protein level might be used as an early diagnostic indicator of this disease.

To further illustrate that NQO1 may be an effective predictor of poor prognosis, the correlation between NQO1 expression and clinicopathological features of cervical, endometrial and ovarian carcinomas was analyzed. We found that high-level expression of the NQO1 protein was significantly correlated with poor differentiation in cervical and EC and not associated with advanced stage. NQO1 overexpression was reported to be associated with high tumor grade in carcinoma of cervix and breast [10, 26], with advanced stage cervical [26], breast [10], colon [27] and liver [28] carcinomas and with nodal metastases [10]. NQO1 overexpression induced tumor cell proliferation via the up-regulation of cyclins [29] and was accompanied by an increase in other antioxidant enzymes, such as HMOX-1 and GST, providing tumors with increased protection against cytotoxic agents allowing for rapid cancer progression [30]. These results indicated that NQO1 played a predictive role in tumor progression and might be useful as a poor prognostic biomarker of cancer.

NQO1 overexpression in tumors but not normal tissue has made it an attractive target for treatment of lung cancer. It is the main activator of quinone-containing alkylating agents such as mitomycins [31]. So that, patients with KRAS mutations may utilize quinone-containing alkylating agents more efficiently due to increased NQO1 expression [11]. On the contrary, loss of NQO1 expression appeared to be candidates for adjuvant chemotherapy in patients with cholangiocarcinoma [32]. Therefore, the role of NQO1 and related inhibitors in chemotherapy appears questionable. A comprehensive similar analysis of the relationship between NQO1 enzyme activity and chemosensitivity in female tract cancer is essential.

In this study, we found that Nrf2 is nuclear, and cytoplasmic marker by immunohistochemistry in cervical and ovarian tissue, while in the endometrium its expression was mainly cytoplasmic with little nuclear expression and these observations were in agreement with previous studies [33-35]. Other reports detect Nrf2 expression mainly in the nucleus [27, 36, 37]. It is well established that oxidative stress is the primary signal that causes cytoplasmic Nrf2 to accumulate within the nucleus [34]. It has been documented that persistent nuclear expression of Nrf2 results in the production of antioxidants that protect cancer cells from reactive oxygen species. Higher concentration of Nrf2 in the nucleus may reflect upstaging of cancer, aggressive tumor behavior and poor clinical outcome [26, 33, 37]. Half of ovarian carcinomas with positive nuclear Nrf2 staining had either Keap1 mutations or absent Keap1 mRNA expression resulting in platinum resistance [38]. So, nuclear Nrf2 expression in cancer cells would have a higher malignant potential. Therefore, it is essential to evaluate nuclear expression of Nrf2.

We found Nrf2 expression was 62%, 64% and 72% in cervical, endometrial and ovarian carcinoma respectively. Previous studies demonstrated that Nrf2 immunoreactivity was frequently detected in various human malignancies, such as intrahepatic chorangiocellular [32], endometrial [33], breast [36], gastric [35, 37], ovarian [38], lung [39], pancreatic [40] and gallbladder [41] carcinomas, and its rate of immunopositivity ranged between 26% and 76% in these studies.

We found increased expression from normal tissue to SIL and cervical carcinoma and then from SIL to carcinoma. The difference between all examined groups was statistically significant. In the endometrium, we found Nrf2 expression was increased from CE to EH to EC, and the difference was statistically significant. Similar results were reported in the endometrium with lower expression of Nrf2 in atypical EH and higher in endometrial cancer [33, 42-44]. Finally in ovarian tissue we found the same results as Nrf2 expression was increased from normal to benign tumors and from benign to borderline tumors and from borderline tumors to carcinomas with a significant difference between the examined groups. This elevated Nrf2 expression may be induced by gonadotropins and sex-steroid hormones, which suggest that these hormones are involved in ovarian cancer development via modulation of Nrf2 signaling. Therefore, its inhibition may represent an effective therapeutic strategy for treatment [45].

Previous studies in different organs reported that Nrf2 expression was more overexpressed in carcinoma than normal and precancerous lesions in pancreatic [34, 40], gastric [35] and breast [36] carcinomas. Nrf2 expression may represent one of the early molecular events in the neoplastic transformation of several tumors.

Regarding association of Nrf2 overexpression and clinicopathological data, we found that its expression was significantly associated with high tumor grade in cervical and EC and with advanced tumor stage in endometrial and ovarian carcinoma and no significant association with the age. Our findings are in accordance with those reported by [27, 35-37, 41]. No differences were noted in age, grade and stage in ovarian carcinoma [38, 45]. Overexpression of Nrf2 in gallbladder adenocarcinoma was correlated with tumor differentiation, staging, metastasis and shorter overall survival [41].

Furthermore, overexpression of Nrf2, a regulator of an intracellular antioxidant response and is negatively regulated by Keap1, may be partially responsible to the aggressive biological behavior and poor clinical outcome due to its known effect of increased resistance to chemotherapeutic drugs as cisplatin in both endometrial and ovarian cancer cells [18, 20, 21]. These findings may also provide an opportunity for therapeutic intervention against chemoresistance via applications of either Nrf2 inhibitors or gene knockdown approaches [33]. Therefore, a new chemotherapeutic protocol that includes antioxidant therapy may be a useful method for solving chemoresistance [37].

Regarding correlation between NQO1 and Nrf2 mean expression in different lesions, we found positive correlations between the two proteins especially in carcinomas. Similar correlations were reported [27]. This Nrf2-NQO1/MRP1 signal pathway may be attributed to the stress response and self-protective effort of the cells during malignant transformation. Considering the role of Nrf2 in regulating genes as NQO1 and MRP1, which act to detoxify drugs or attenuate drug-induced oxidative stress, it is possible that highly expressed nuclear Nrf2 plays a role in increasing treatment resistance and results in short survival [27]. To the best of our knowledge, this was the first study examining NQO1 and Nrf2 in cervical, endometrial and ovarian tissue and the change of their expression in different lesions in each tissue type.

### Conclusions

NQO1 and Nrf2 play a key role in the progression of female tract tumors, and high level of both proteins were strongly associated with high grade and advanced stage. The high proportion of NQO1 expression suggests that NQO1 may be a significant biomarker and a potential therapeutic target for carcinoma patients. Nrf2 expression in cancer may be useful for evaluation of biological malignant potential. Overall, our present work implies that NQO1 and Nrf2 might be new biomarkers for early diagnosis and tumorigenesis in patients with tumors of female genital tract.

## References

[1] Weiderpass E, Labreche F (2012). Malignant tumors of the female reproductive system. Saf Health Work.

[2] Ferlay J, Shin HR, Bray F, Forman D, Mathers C, Parkin DM (2010). Cancer incidence and mortality worldwide: IARC CancerBase No. 10. Lyon (France). International Agency for Research on Cancer.

[3] Merritt MA, Cramer DW (2010). Molecular pathogenesis of endometrial and ovarian cancer. Cancer Biomark.

[4] Ibrahim AS, Khaled HM, Mikhail NN, Baraka H, Kamel H (2014). Cancer incidence in egypt: results of the national population-based cancer registry program. J Cancer Epidemiol.

[5] Lajin B, Alachkar A (2013). The NQO1 polymorphism C609T (Pro187Ser) and cancer susceptibility: a comprehensive meta-analysis. Br J Cancer.

[6] Hu X, Zhang Z, Ma D, Huettner PC, Massad LS, Nguyen L, Borecki I (2010). TP53, MDM2, NQO1, and susceptibility to cervical cancer. Cancer Epidemiol Biomarkers Prev.

[7] Su XL, Yan MR, Yang L (2012). NQO1 C609T polymorphism correlated to colon cancer risk in farmers from western region of Inner Mongolia. Chin J Cancer Res.

[8] Awadallah NS, Dehn D, Shah RJ, Russell Nash S, Chen YK, Ross D, Bentz JS (2008). NQO1 expression in pancreatic cancer and its potential use as a biomarker. Appl Immunohistochem Mol Morphol.

[9] Buranrat B, Chau-in S, Prawan A, Puapairoj A, Zeekpudsa P, Kukongviriyapan V (2012). NQO1 expression correlates with cholangiocarcinoma prognosis. Asian Pac J Cancer Prev.

[10] Yang Y, Zhang Y, Wu Q, Cui X, Lin Z, Liu S, Chen L (2014). Clinical implications of high NQO1 expression in breast cancers. J Exp Clin Cancer Res.

[11] Yilmaz A, Mohamed N, Patterson KA, Tang Y, Shilo K, Villalona-Calero MA, Davis ME (2014). Increased NQO1 but not c-MET and survivin expression in non-small cell lung carcinoma with KRAS mutations. Int J Environ Res Public Health.

[12] Zappa F, Ward T, Pedrinis E, Butler J, McGown A (2003). NAD(P)H: quinone oxidoreductase 1 expression in kidney podocytes. J Histochem Cytochem.

[13] Marjani HA, Biramijamal F, Rakhshani N, Hossein-Nezhad A, Malekzadeh R (2010). Investigation of NQO1 genetic polymorphism, NQO1 gene expression and PAH-DNA adducts in ESCC. A case-control study from Iran. Genet Mol Res.

[14] Bey EA, Reinicke KE, Srougi MC, Varnes M, Anderson VE, Pink JJ, Li LS (2013). Catalase abrogates beta-lapachone-induced PARP1 hyperactivation-directed programmed necrosis in NQO1-positive breast cancers. Mol Cancer Ther.

[15] Motohashi H, Yamamoto M (2004). Nrf2-Keap1 defines a physiologically important stress response mechanism. Trends Mol Med.

[16] Zhang DD (2006). Mechanistic studies of the Nrf2-Keap1 signaling pathway. Drug Metab Rev.

[17] Kwak MK, Wakabayashi N, Itoh K, Motohashi H, Yamamoto M, Kensler TW (2003). Modulation of gene expression by cancer chemopreventive dithiolethiones through the Keap1-Nrf2 pathway. Identification of novel gene clusters for cell survival. J Biol Chem.

[18] Jiang T, Huang Z, Lin Y, Zhang Z, Fang D, Zhang DD (2010). The protective role of Nrf2 in streptozotocin-induced diabetic nephropathy. Diabetes.

[19] Kobayashi A, Kang MI, Okawa H, Ohtsuji M, Zenke Y, Chiba T, Igarashi K (2004). Oxidative stress sensor Keap1 functions as an adaptor for Cul3-based E3 ligase to regulate proteasomal degradation of Nrf2. Mol Cell Biol.

[20] Cho JM, Manandhar S, Lee HR, Park HM, Kwak MK (2008). Role of the Nrf2-antioxidant system in cytotoxicity mediated by anticancer cisplatin: implication to cancer cell resistance. Cancer Lett.

[21] Wang XJ, Sun Z, Villeneuve NF, Zhang S, Zhao F, Li Y, Chen W (2008). Nrf2 enhances resistance of cancer cells to chemotherapeutic drugs, the dark side of Nrf2. Carcinogenesis.

[22] Clarke BA, Gilks B (2011). Ovarian Carcinoma: Recent Developments in Classification of Tumour Histological Subtype. Canadian Journal of Pathology.

[23] Edge SB, Byrd DR, Compton CC, Fritz AG, Greene FL, Trotti A (2010). Ovary and primary peritoneal carcinoma. AJCC Cancer Staging Manual.

[24] Lin L, Qin Y, Jin T, Liu S, Zhang S, Shen X, Lin Z (2014). Significance of NQO1 overexpression for prognostic evaluation of gastric adenocarcinoma. Exp Mol Pathol.

[25] Soucek P, Susova S, Mohelnikova-Duchonova B, Gromadzinska J, Moraviec-Sztandera A, Vodicka P, Vodickova L (2010). Polymorphisms in metabolizing enzymes and the risk of head and neck squamous cell carcinoma in the Slavic population of the central Europe. Neoplasma.

[26] Ma Y, Kong J, Yan G, Ren X, Jin D, Jin T, Lin L (2014). NQO1 overexpression is associated with poor prognosis in squamous cell carcinoma of the uterine cervix. BMC Cancer.

[27] Ji L, Wei Y, Jiang T, Wang S (2014). Correlation of Nrf2, NQO1, MRP1, cmyc and p53 in colorectal cancer and their relationships to clinicopathologic features and survival. Int J Clin Exp Pathol.

[28] Chiu MM, Ko YJ, Tsou AP, Chau GY, Chau YP (2009). Analysis of NQO1 polymorphisms and p53 protein expression in patients with hepatocellular carcinoma. Histol Histopathol.

[29] Garate M, Wani AA, Li G (2010). The NAD(P)H:Quinone Oxidoreductase 1 induces cell cycle progression and proliferation of melanoma cells. Free Radic Biol Med.

[30] Lau A, Villeneuve NF, Sun Z, Wong PK, Zhang DD (2008). Dual roles of Nrf2 in cancer. Pharmacol Res.

[31] Beall HD, Winski SI (2000). Mechanisms of action of quinone-containing alkylating agents. I: NQO1-directed drug development. Front Biosci.

[32] Wakai T, Shirai Y, Sakata J, Matsuda Y, Korita PV, Takamura M, Ajioka Y (2011). Prognostic significance of NQO1 expression in intrahepatic cholangiocarcinoma. Int J Clin Exp Pathol.

[33] Chen N, Yi X, Abushahin N, Pang S, Zhang D, Kong B, Zheng W (2010). Nrf2 expression in endometrial serous carcinomas and its precancers. Int J Clin Exp Pathol.

[34] Lister A, Nedjadi T, Kitteringham NR, Campbell F, Costello E, Lloyd B, Copple IM (2011). Nrf2 is overexpressed in pancreatic cancer: implications for cell proliferation and therapy. Mol Cancer.

[35] Hu XF, Yao J, Gao SG, Wang XS, Peng XQ, Yang YT, Feng XS (2013). Nrf2 overexpression predicts prognosis and 5-FU resistance in gastric cancer. Asian Pac J Cancer Prev.

[36] Onodera Y, Motohashi H, Takagi K, Miki Y, Shibahara Y, Watanabe M, Ishida T (2014). NRF2 immunolocalization in human breast cancer patients as a prognostic factor. Endocr Relat Cancer.

[37] Kawasaki Y, Ishigami S, Arigami T, Uenosono Y, Yanagita S, Uchikado Y, Kita Y (2015). Clinicopathological significance of nuclear factor (erythroid-2)-related factor 2 (Nrf2) expression in gastric cancer. BMC Cancer.

[38] Konstantinopoulos PA, Spentzos D, Fountzilas E, Francoeur N, Sanisetty S, Grammatikos AP, Hecht JL (2011). Keap1 mutations and Nrf2 pathway activation in epithelial ovarian cancer. Cancer Res.

[39] Solis LM, Behrens C, Dong W, Suraokar M, Ozburn NC, Moran CA, Corvalan AH (2010). Nrf2 and Keap1 abnormalities in non-small cell lung carcinoma and association with clinicopathologic features. Clin Cancer Res.

[40] Hong YB, Kang HJ, Kwon SY, Kim HJ, Kwon KY, Cho CH, Lee JM (2010). Nuclear factor (erythroid-derived 2)-like 2 regulates drug resistance in pancreatic cancer cells. Pancreas.

[41] Wang J, Zhang M, Zhang L (2010). Correlation of Nrf2, HO-1, and MRP3 in gallbladder cancer and their relationships to clinicopathologic features and survival. J Surg Res.

[42] Liang SX, Chambers SK, Cheng L, Zhang S, Zhou Y, Zheng W (2004). Endometrial glandular dysplasia: a putative precursor lesion of uterine papillary serous carcinoma. Part II: molecular features. Int J Surg Pathol.

[43] Fadare O, Liang SX, Ulukus EC, Chambers SK, Zheng W (2006). Precursors of endometrial clear cell carcinoma. Am J Surg Pathol.

[44] Yi X, Zheng W (2008). Endometrial glandular dysplasia and endometrial intraepithelial neoplasia. Curr Opin Obstet Gynecol.

[45] Liao H, Zhou Q, Zhang Z, Wang Q, Sun Y, Yi X, Feng Y (2012). NRF2 is overexpressed in ovarian epithelial carcinoma and is regulated by gonadotrophin and sex-steroid hormones. Oncol Rep.

